# The Parameters of Transcutaneous Electrical Nerve Stimulation Are Critical to Its Regenerative Effects When Applied Just after a Sciatic Crush Lesion in Mice

**DOI:** 10.1155/2014/572949

**Published:** 2014-07-24

**Authors:** Diana Cavalcante Miranda de Assis, Êmyle Martins Lima, Bruno Teixeira Goes, João Zugaib Cavalcanti, Alaí Barbosa Paixão, Marcos André Vannier-Santos, Ana Maria Blanco Martinez, Abrahão Fontes Baptista

**Affiliations:** ^1^Post-Graduation Program in Medicine and Health, Faculty of Medicine, Federal University of Bahia, Rua Augusto Viana, Canela, 40110-060 Salvador, BA, Brazil; ^2^Functional Electrostimulation Laboratory, Department of Biomorphology, Federal University of Bahia, Avenida Reitor Miguel Calmon, S/N, Vale do Canela, 40110-902 Salvador, BA, Brazil; ^3^Gonçalo Moniz Research Center, Oswaldo Cruz Foundation (FIOCRUZ), Rua Waldemar Falcão, Candea, INCT-INPeTAm/MCT-CNPq, 40296-710 Salvador, BA, Brazil; ^4^Biotechnology and Bioprospection Nucleus (NBBio), Bahian School of Medicine and Human Health, Rua Frei Henrique, 8 Nazaré, 40.050-420 Salvador, BA, Brazil; ^5^Physiology and Synaptic Plasticity Laboratory, Department of Physiology, Faculty of Medicine of Ribeirão Preto, University of São Paulo, São Paulo, SP, Brazil; ^6^IBCCF, Cidade Universitária, Avenida Pedro Calmon, 21941-901 Rio de Janeiro, RJ, Brazil; ^7^Neuroregeneration and Repair Laboratory, Federal University of Rio de Janeiro-UFRJ, Avenida Pedro Calmon, Cidade Universitária, 21941-901 Rio de Janeiro, RJ, Brazil

## Abstract

We investigated the effect of two frequencies of transcutaneous electrical nerve stimulation (TENS) applied immediately after lesion on peripheral nerve regeneration after a mouse sciatic crush injury. The animals were anesthetized and subjected to crushing of the right sciatic nerve and then separated into three groups: nontreated, Low-TENS (4 Hz), and High-TENS (100 Hz). The animals of Low- and High-TENS groups were stimulated for 2 h immediately after the surgical procedure, while the nontreated group was only positioned for the same period. After five weeks the animals were euthanized, and the nerves dissected bilaterally for histological and histomorphometric analysis. Histological assessment by light and electron microscopy showed that High-TENS and nontreated nerves had a similar profile, with extensive signs of degeneration. Conversely, Low-TENS led to increased regeneration, displaying histological aspects similar to control nerves. High-TENS also led to decreased density of fibers in the range of 6–12 *μ*m diameter and decreased fiber diameter and myelin area in the range of 0–2 *μ*m diameter. These findings suggest that High-TENS applied just after a peripheral nerve crush may be deleterious for regeneration, whereas Low-TENS may increase nerve regeneration capacity.

## 1. Introduction

Despite the ability to regenerate, the functional recovery of the peripheral nervous system is often poor. Morphological and physiological processes determine the restoring of the electrical activity of neurons involved in the injury, and these in turn are required for the complete recovery of motor function after nerve injury [1]. Traumas, blocking axonal transport, and chemical toxicity are some of the insults that may lead to impairment of normal neuronal function [2].

There are several experimental strategies used to overcome the limitations associated with the progressive loss of the regenerative capacity, which is usually seen in lesions occurring far from the target organ; these strategies include electrical stimulation, which can modulate the molecular and cellular activity involved in the regenerative process [3]. However, the parameters of electrical stimulation appear to be critical for obtaining the desired results. Numerous studies have shown that low-frequency electrical stimulation applied by means of electrodes in direct contact with the nerve after injury and surgical repair may improve nerve regeneration and accelerate reinnervation of the target organs [4], increase nerve fiber density and diameter [5], enhance myelination and angiogenesis [6], and increase nerve growth factor (NGF) [7] and brain-derived neurotrophic factor (BDNF) release and expression [8].

The use of surface electrodes may comprise an alternative to direct stimulation of the nerve, considering that it involves lower risks and a simpler methodology of application, and can be used for a longer period, especially when combined with biphasic electric currents. However, previous results have shown that transcutaneous electrical nerve stimulation (TENS) applied for an extended period, that is, five weeks after an experimental crush lesion to the sciatic nerve, led to inhibition of regeneration in mice [9]. Nevertheless, it has been demonstrated that an improvement in peripheral nerve regeneration can be achieved when a low-frequency electrical stimulation is applied immediately after injury, but the number of sessions is not determinant when stimulation is performed earlier [10].

As TENS may be a simple and useful method to apply electrical currents to influence peripheral nerve regeneration, it is mandatory to elucidate which are the most effective procedures for stimulating nerve growth/regeneration after lesion. Therefore, the objective of this study was to assess the influence of early application of High- and Low-frequency TENS on mice peripheral nerve regeneration after sciatic crush injury.

## 2. Materials and Methods

This study involved 15 Swiss mice (*Mus musculus*), weighing 35–48 g. The sample size was defined based on our previous study [9]. All procedures were approved by the Committee for Animal Experimentation Ethics of the Bahian School of Medicine and Public Health, under the protocol 003/2009. The animals were housed in individual cages with food and water* ad libitum* and a 12 : 12 h light/dark cycle.

### 2.1. Surgery

The animals were anesthetized with ketamine (10 mg/kg) and xylazine (100 mg/kg) and then subjected to asepsis and trichotomy of the rear right limb. After a longitudinal incision, the right sciatic nerve was exposed, isolated from adjacent tissues, and crushed just distal to the sciatic notch with needle holder forceps maintained for 30 seconds on the first lock. This method was the same as that we used in our previous study and has demonstrated to promote a clear and standardized lesion to the sciatic nerve, with total loss of function after seven days of injury [9]. Muscle and skin were sutured using 4.0 absorbable and nonabsorbable sutures, respectively. During the experimental period, signs of distress due to nerve injury, such as autotomy, weight loss, and general hypomobility, were monitored.

### 2.2. Electrical Stimulation

The animals were stimulated for two hours after the surgical procedure. During stimulation they were lightly anesthetized with a mixture of halothane and oxygen (1 L/min), administered through a vaporizer (Takaoka, USA). Electrical stimulation was delivered through clinical biphasic pulse generator (TENS vif 962, QUARK Medical, Brazil), previously calibrated for the study. Electrical current was transmitted by two silicon-carbon electrodes (1.5 cm^2^ area), using carbopol gel. The electrodes were placed along the incision, with a distance of 2 cm between them. The electrical parameters were based on our previous study [9].

The animals were divided into three groups:

1. nontreated: animals subjected to the sciatic injury, standard protocol for anesthesia and positioning, but no electrical stimulation (*n* = 5);
2. Low-TENS: 4 Hz frequency, modulated in 2 Hz bursts, with amplitude just within the motor threshold (*n* = 5);
3. High-TENS: 100 Hz frequency, with amplitude just below the motor threshold (*n* = 5).

For histomorphometric analysis, the left nerves (uninjured and unstimulated) were considered as controls. The nerves on the right side (injured) were grouped into nontreated, Low-TENS, and High-TENS.

### 2.3. Histological and Histomorphometric Assessment

On the 35th day after lesion, the animals were deeply anesthetized and euthanized by transcardiac perfusion with fixative solution (4% paraformaldehyde and 2% glutaraldehyde in 0.1 M sodium cacodylate buffer, pH 7.4, 50 mL/animal). The sciatic nerves ipsilateral to the lesion were harvested, and a 2 mm segment, 3 mm distal to the lesion site, was dissected. Contralateral nerves were also dissected, and a 2 mm segment was collected from the equivalent portion of the lesioned nerve. The segments were postfixed in osmium tetroxide, dehydrated in increasing concentrations of acetone (30–100%), infiltrated in acetone and resin, and plastic-embedded. Transverse sections 0.5 *μ*m (semithin) and 70 nm thick (ultrathin) were obtained using an ultramicrotome (Reichert Jung, USA). The semithin sections were stained with a mixture of 1 : 1 toluidine blue and azur 2. Images were acquired with a light microscope (Olympus BX 51) connected to a digital camera (Olympus Q color 5).

For the ultrastructural analysis, 70 nm thick sections were stained with 1% uranyl acetate and 3% lead citrate for five minutes and observed through a transmission electron microscope (Zeiss EM 109) equipped with an image acquisition system (MegaView II, Analysis-Imaging-System). For qualitative assessment of the overall condition of the nerve we used 1,000x (light microscopy) and 7,000x (electron microscopy) magnification images of the four groups of nerves.

A magnification of 3,000x was used to measure diameter of myelinated fibers, stratified at 0–1.99 (0–2), 2–5.99 (2–6), and 6–12 *μ*m diameters. Then density, axon diameter, myelin area, and *G* ratio (axon diameter/fiber diameter) were evaluated in each stratum. A magnification of 7,000x was used for morphological assessment and measurement of the densities and diameters of nonmyelinated fibers and the density of Schwann cell (SC) nuclei. Densities were calculated by dividing the number of cells by the total area of 10 systematically chosen fields.

The independent variable for all groups was the use of TENS. The dependent variables were derived from histomorphometry. All descriptive data were presented as median and 25/75 quartiles. Nonpaired inferences were performed by Kruskal-Wallis test associated with Dunn's post hoc analysis. Where differences were not detectable by this post hoc analysis, Mann-Whitney comparisons were performed. An alpha value of 5% (*P* < 0.05) was considered significant. The analyses were carried out using the statistical package Graph Pad Prism 6.0.

## 3. Results

### 3.1. Histological Assessment

**Figure 1:**
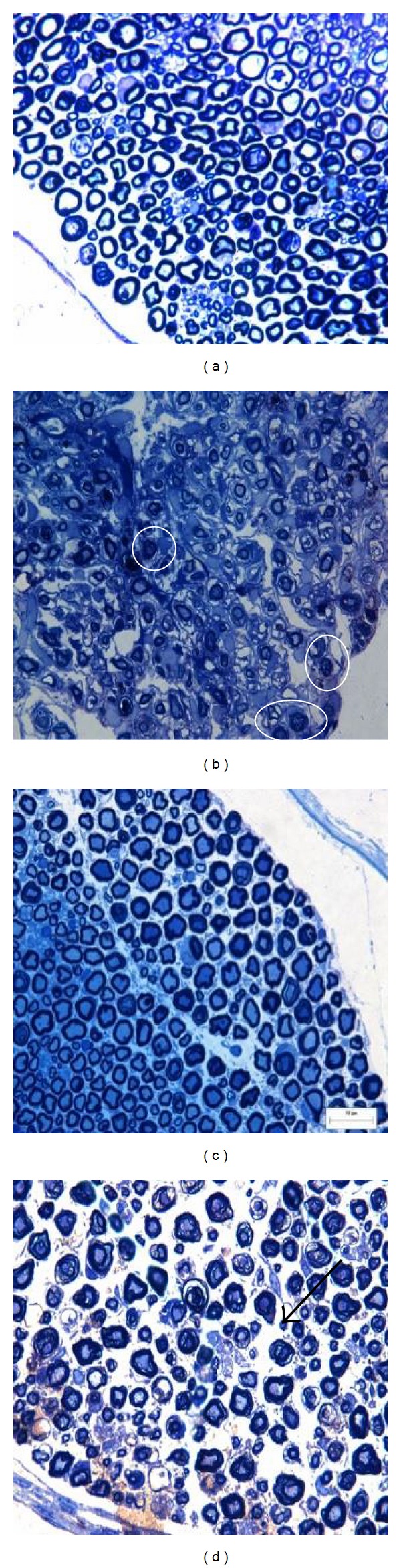
Semithin transverse sciatic nerve sections (1,000x magnification). (a) Control group (without nerve injury or stimulation) showing a compacted distribution of nerves fibers, and no signs of lesion; (b) nontreated group (unstimulated injured nerve) showing lower fiber density than normal group, and many axons with dark axoplasm (circles); (c) Low-TENS group (damaged nerve with low-frequency electrical stimulation) displaying fiber diameter, density, and distribution similar to the control group; (d) High-TENS group (damaged nerve with high-frequency stimulation) showing markedly increased endoneurial space (black arrow), which may represent edema. Magnification bar: 10 *μ*m.

Morphological changes were observed in the images acquired by light and electron microscopy. In light microscopy images, the histological appearance of the nerves of the control and Low-TENS groups was generally better than the nerves of the nontreated or High-TENS groups (Figure 1), which showed more frequently axons with dark axoplasm (Figure 1(c)), one of the signs of axon degeneration. The nerves of the Low-TENS group showed myelinated nerve fibers with diameters similar to those of the control nerves (Figure 1). When comparing the Low- and High-TENS groups, the latter presented a worse aspect, with more frequent dark axoplasm axons and fewer myelinated nerve fibers (Figures 1(b) and 1(c)).

**Figure 2:**
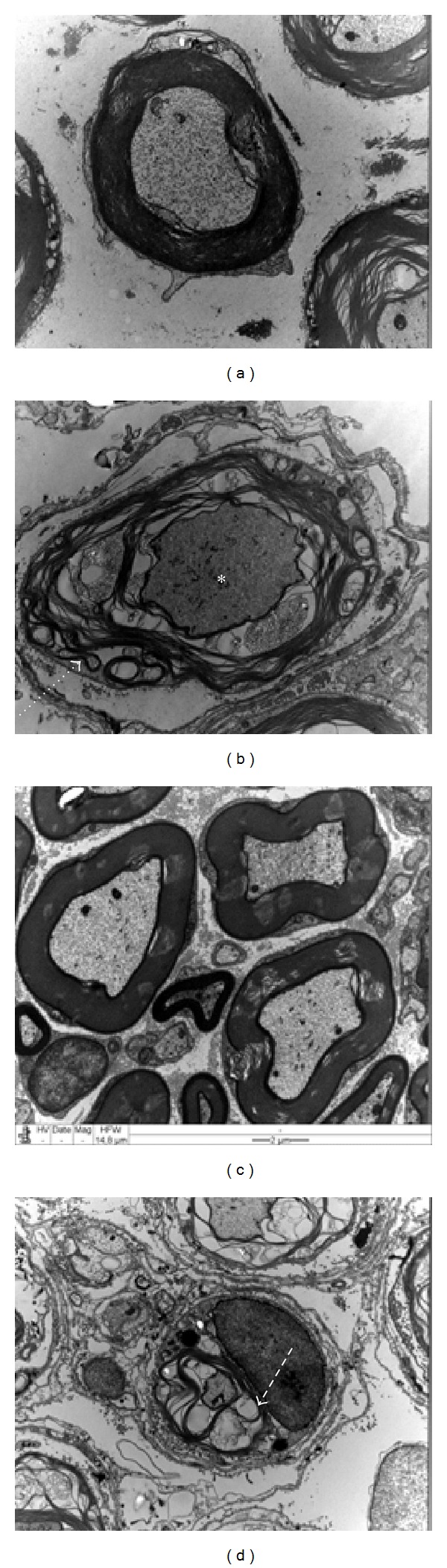
Sciatic nerve ultrathin transverse sections (7,000x magnification). (a) Control group (intact unstimulated nerve) showing large myelinated fibers; (b) nontreated group (unstimulated injured nerve) showing a fiber with dissolute axoplasm (asterisk), myelin disruption (dashed, white arrow). (c) Low-TENS group (damaged nerve with low-frequency electrical stimulation), where myelinated fibers were well preserved, showing a normal aspect; (d) High-TENS group (damaged nerve with high-frequency stimulation), with a fiber presenting myelin disruption culminating in complete demyelination (dashed, white arrow). Magnification bar: 2 *μ*m.

Regarding the nerve morphology shown in 7,000x magnification electromicrographs, it was observed that the nerves in the nontreated group showed signs of axonopathy, characterized by the presence of a nonspecific laminar membrane structure, numerous macrophages, axoplasm dissolution, regenerative cluster disorganization, and denervated Schwann cell bands. Myelin sheaths showed characteristic fragmentation (Figure 2). In the High-TENS nerves it was possible to observe signs of extensive demyelination and many unmyelinated/remyelinated fibers (Figure 2(d)). The Low-TENS nerves showed a better aspect, with continuity of the basal lamina, a higher density of myelinated fibers (Figure 2(c)), and several grouped bands of unmyelinated fibers. Denervated SC could be found in both groups.

### 3.2. Morphometric Assessment

**Figure 3:**
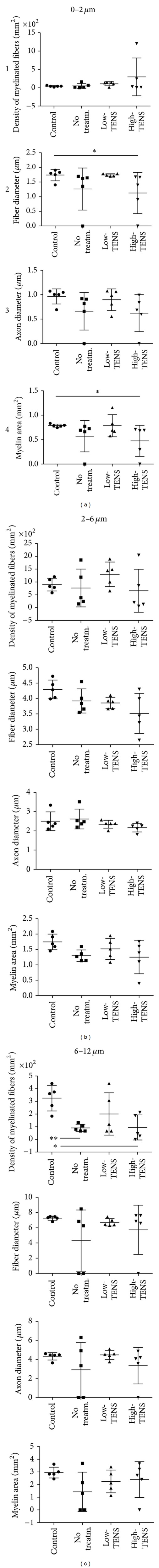
Morphometric data from myelinated fibers grouped into three diameter ranges (0–2, 2–6, and 6–12 *μ*m). Samples were analyzed in myelinated fiber density (1), diameter (2), and axon diameter (3). Nontreated and High-TENS nerves presented fewer fibers than control or Low-TENS in the range of 6–12 *μ*m (1(c)). High-TENS nerves also presented lower diameter fibers (2(a)) and less myelin area (4(a)) than the other groups in the range of 0–2 *μ*m. Data are presented as median and 25–75 quartiles. *P < 0.05, **P < 0.01.

The morphometric analysis was performed using the images obtained by an electron microscope. High-TENS and nontreated nerves showed fewer fibers in the 6–12 *μ*m strata, as compared to control (Kruskal-Wallis = 8.18, *P* < 0.05; control × nontreated, *P* < 0.01; control × High-TENS, *P* < 0.05). In the same strata, nerves stimulated with Low-TENS did not show differences from control nerves (*P* = 0.25) (Figures 3(1c)).

**Figure 4:**
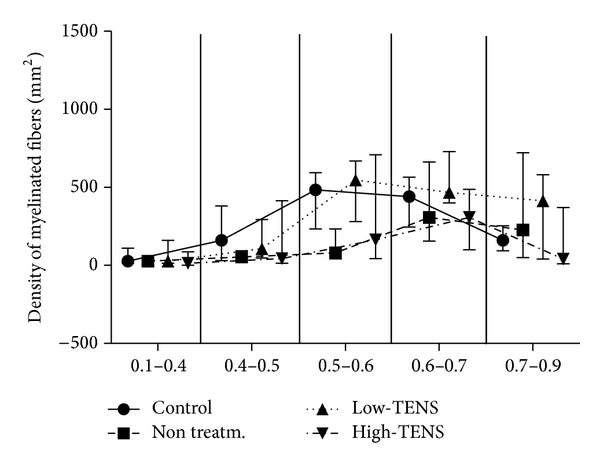
*G* ratio stratified by ranges. *G* coefficient was obtained by dividing the axon by fiber diameter. Note that the nerves in the control and Low-TENS groups showed a peak fiber density in the range of 0.5–0.6, while nontreated High-TENS showed this peak at the 0.6–0.7 range. Data are presented as median and 25–75 quartiles.

High-TENS nerves also presented lower fiber diameter (Kruskal-Wallis = 11.71, *P* < 0.01, Dunn *P* < 0.05) (Figure 2(a)) and lower myelin area (Kruskal-Wallis = 7.96, *P* < 0.05, Dunn *P* < 0.05) (Figures 3(2a) and 3(4a)). *G* ratio analysis confirmed similar trends between control and Low-TENS and nontreated and High-TENS nerves. The former presented a peak of myelinated fibers in the range of 0.5–0.6, whereas the latter ones had this peak in the range of 0.6–0.7, presenting a worse myelin/axon ratio (Figure 4). All other myelinated fiber measures, nonmyelinated fiber densities (Kruskal-Wallis = 1.78, *P* = 0.64), and Schwann cells density (Kruskal-Wallis = 1.43, *P* = 0.70) were not different among groups.

## 4. Discussion

In this study, we observed that animals treated with Low-TENS showed evidence of accelerated sciatic nerve regeneration. This effect was not found in animals treated with High-TENS. We noticed that the nerves treated with Low-TENS showed improved qualitative and quantitative parameters in relation to High-TENS. Nerves undergoing Low-TENS did not exhibit signs of degeneration seen in groups without treatment or treated with High-TENS, such as dark axoplasm, high density of macrophages, axoplasmic dissolution, nonspecific laminar structure of the membrane, and regenerative clusters disintegration. On the 35th day after injury, the nerves of animals treated with Low-TENS exhibited density, fiber diameter, and degree of myelination similar to the control group. Conversely, treatment with High-TENS was ineffective and led to failed regeneration, similar to the untreated group. In this context, we highlight a lower density of large diameter fibers (6–12 *μ*m) and a smaller diameter and myelin content of low diameter fibers (0–2 *μ*m).

The stimulation protocol we chose for this study meant to reproduce the clinical application of TENS, which is primarily used for pain relief in the low- (1–10 Hz) and high-frequency (50–100 Hz) ranges [11]. As it is frequently used to treat neuropathic pain conditions, which may be associated with Wallerian degeneration, we ought to assess the effect of electrical stimulation in these frequencies, applied just after the lesion, on peripheral nerve regeneration. In addition, few studies have investigated the effect of alternating electrical currents as a way to improve nerve regeneration [9, 12], and very little is known about the effect of this type of electrotherapy on histomorphometric characteristics of nerves after crush injury [13], since monophasic currents are the most commonly used strategy to stimulate peripheral nerve regeneration [13, 14]. Particularly, we used surface electrodes to the current application, which is also different from other works in this area. This poses the advantage of noninvasive procedures, decreasing complication risks and costs of the intervention.

Our results corroborate previous investigations, mainly with invasive electrodes, showing that low-frequency electrical stimulation applied immediately after a peripheral nerve injury accelerates peripheral nerve regeneration [4, 15–17]. In general, similar stimulation protocols are efficient in restoring biochemical, biophysical, morphological, and functional aspects of peripheral nerve after different types of injury and ways to manage the electrical current [18–21]. The morphometric parameters investigated suggest that Low-TENS might positively influence the conditions of the microenvironment for axonal regeneration [22], which remains to be investigated.

Beirowski et al. [23] demonstrated with accurate methods that degeneration after crush injury tends to spread retrogradely towards the cell body. Accordingly, a possible effect of our intervention with Low-TENS was mitigating this degeneration and preserving the cellular machinery that enables regeneration. Moreover, successful regeneration is based, in the first instance, on the survivability of affected axons [24]. Although some mechanisms of this phenomenon remain unclear, the level of intracellular (axoplasmic) Ca^2+^ appears to trigger a number of signaling cascades in neurons and Schwann cells [7, 25, 26]. Accurate elevations in intracellular levels of this ion are essential to gene and protein expression and synthesis involved in regeneration [27].

Immediately after an injury, the neuron begins to show high-frequency burst activity, which increases intracellular Ca^2+^ and eventually induces apoptosis [24]. From this perspective, Low-TENS may have modulated the levels of intracellular Ca^2+^, and consequently the activity of the injured neurons through voltage-dependent channels. It is possible that the low-frequency stimulation decreased the firing frequency of these neurons and adequately stimulated the production of immediate gene expression of neurotrophic (BDNF, NGF, and VEGF) and other transcription factors, which can mediate the improvement of the histomorphometric parameters [26, 28, 29]. In contrast, 100 Hz High-TENS may have increased neuronal firing to a toxic degree and did not demonstrate a potential to increase regeneration.

These results were confirmed by *G* ratio indices, where Low-TENS nerves behaved always similar to uninjured nerves. As this is an indirect measure of functional nerve transmission, our data cannot distinguish between the influences of Low-TENS in sensory and motor function. However, several studies showed a positive correlation between improvement of morphological and functional components following peripheral nerve injury [30–32]. In fact, the accuracy of a sensory motor function depends on the integrity of its morphological and functional components.

There is considerable controversy about the best protocol of electrical stimulation for peripheral nerve regeneration [8, 33, 34]. In a previous study we have shown that both Low- and High-TENS applied five days per week, for five weeks, led to inhibited nerve regeneration in a sciatic crush model [9]. Although our data are not conclusive, they provide evidence that Low-TENS used early after injury, just once, can accelerate peripheral nerve regeneration. Evidence indicates that early and brief protocols favor the regenerative process of peripheral nerves [17, 18, 32]. Furthermore, frequency of the electric current seems to be another important factor to improve nerve regeneration, with low frequencies being preferable [19, 20, 31].

In this sense, our data support the idea that peripheral nerve regeneration efficiency is increased by early, brief, and low-frequency electrical stimulation. A potential limitation of our study was the method of crushing the sciatic nerve with a needle holder. The variability of this method, although not big, may be avoided in the future by most precise ones. We suggest that this protocol using low-frequency alternating currents and surface electrodes is investigated with other methodologies, including their effects on intracellular calcium concentration and mechanisms of neuroprotection. Also, nerve transections should be studied, as they are challenging lesions. The use of TENS to promote nerve regeneration may be a low-risk, inexpensive, and practical way of improving nerve regeneration in the future.
